# Role of Stereotactic Radiosurgery in the Management of Multiple Metastases in the Region of the Motor Cortex: Long-term Survival in Three Cases

**DOI:** 10.7759/cureus.1946

**Published:** 2017-12-14

**Authors:** Rimal H Dossani, Devi P Patra, Hai Sun, Anil Nanda, Federico Ampil

**Affiliations:** 1 Department of Neurosurgery, LSU Health Sciences Center Shreveport; 2 Department of Radiation Oncology, LSU Health Sciences Center Shreveport

**Keywords:** stereotactic radiosurgery, multiple brain metastases, motor cortex

## Abstract

The management of patients with multiple brain metastases, in contrast to those with solitary metastases, continues to evolve. Recent evidence suggests that aggressive microsurgical and radiosurgical management of patients with multiple brain metastases may lead to improved survival and quality of life. The three cases discussed in this report are examples of patients with multiple brain metastases who had excellent outcomes following treatment with microsurgical and radiosurgical approaches. A common feature of each patient is the presence of multiple metastases in the region of the motor cortex. The rationale for this selection is to demonstrate that aggressive management can have a favorable outcome despite the presence of multiple metastases in eloquent regions of the brain.

## Introduction

Intracranial metastases are the most common type of brain tumor, occurring in 20% to 30% of patients with systemic cancer, of whom roughly 50% have multiple brain metastases [1]. In one study, the median survival time (MST) from the time of presentation was five months in patients with solitary metastasis and three months in patients with multiple metastases [2]. For patients with solitary metastasis, landmark randomized clinical trials (RCTs) have shown improvement in survival after treatment with stereotactic radiosurgery (SRS) and microsurgical resection [3-4]. Patchell, et al. demonstrated improved survival in patients with solitary brain metastasis who underwent surgery and whole brain radiation therapy (WBRT) (MST 9.2 months) versus WBRT alone (MST 3.5 months) [3]. Andrews, et al. showed a survival benefit for WBRT + SRS (MST 6.5 months) versus WBRT alone (MST 4.9 months) in patients with solitary brain metastasis [4].

The management of multiple metastases, unlike that of solitary brain metastasis, is not as straightforward. Despite the multifocal nature of multiple brain metastases, various studies have demonstrated the utility of microsurgical resection and SRS in such patients. In a retrospective series, Bindal, et al. demonstrated no difference in survival (MST 14 months) between patients who underwent resection of solitary metastatic lesions versus resection of all metastatic lesions [5]. In a prospective observational study, Yamamoto, et al. demonstrated that SRS alone as the initial treatment for patients with 5-10 brain metastases is non-inferior to that of patients with 2-4 metastases, with MST of 10.8 months for both groups [6]. These studies suggest that aggressive microsurgical and radiosurgical management improve the outcomes of patients with multiple brain metastases.

The heterogeneity associated with multiple brain metastases demands innovative treatment approaches and requires that each patient be treated on a case-by-case basis. In this report, we present three cases demonstrating exceptional outcomes after multimodality treatment with SRS and microsurgical resection. A common feature of each patient is the presence of multiple metastases in the region of the motor cortex. The rationale for this selection is to demonstrate that aggressive management can have a favorable outcome despite the presence of multiple metastases in eloquent regions of the brain. The evidence-based reasoning for the treatment of each patient is presented in the discussion.

## Case presentation

**Figure 1:**
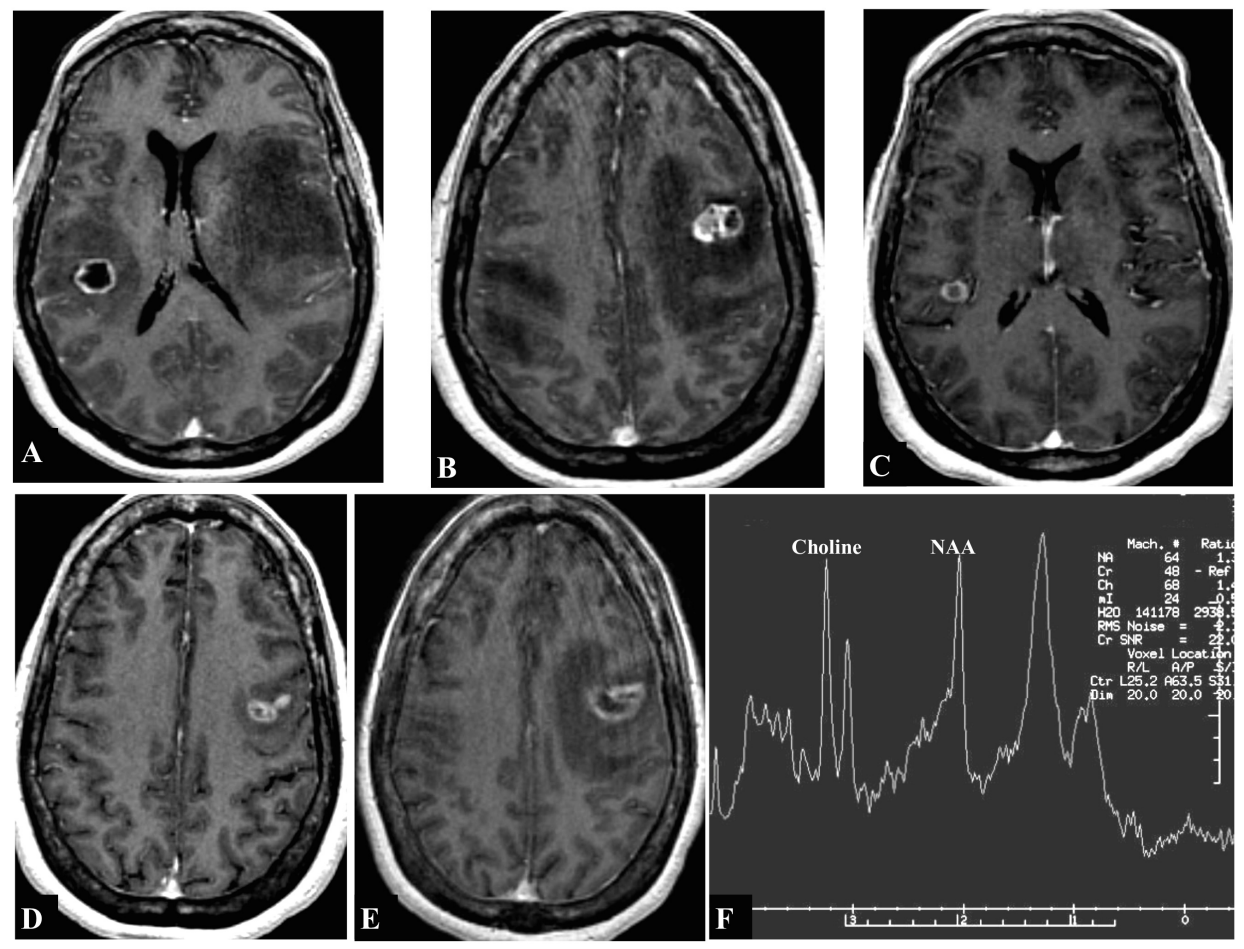
T1-weighted magnetic resonance imaging (MRI) with contrast showing contrast-enhancing lesions Lesions seen in the (A) right precentral gyrus (1.35 cm in diameter), (B) left middle frontal gyrus (1.53 cm), (C) right superior temporal gyrus (<1 cm), (D) left middle frontal gyrus, recurrence, and (E) left middle frontal gyrus, radiation necrosis. (F) MRI spectroscopy of the left middle frontal gyrus lesion was consistent with radiation necrosis (Choline > NAA).

A. Case 1:

The patient is a 60-year-old female who presented with right upper extremity weakness (4+/5 strength). Magnetic resonance Imaging (MRI) of the brain with contrast demonstrated two lesions: one in the right precentral gyrus (1.35 cm) as seen in Figure 1A and one in the left middle frontal gyrus (1.53 cm) as seen in Figure 1B, both with peritumoral edema around the motor cortex. A biopsy of left upper lung lobe lesion was consistent with non-small cell lung cancer (adenocarcinoma). Both brain lesions were treated with Gamma Knife (GK) SRS alone (24 Gray (Gy) at the 50% isodose line to each lesion). A follow-up MRI obtained seven months after GK demonstrated complete resolution of the treated lesions, but also demonstrated a new, less than 1 cm lesion in the right superior temporal gyrus, which was treated with GK (15Gy at the 50% isodose line) (Figure 1C). A follow-up MRI obtained nine months after second GK treatment demonstrated excellent tumor control for lesions in the right precentral gyrus and the right superior temporal gyrus, but showed recurrence of the tumor in the left middle frontal gyrus, which was retreated with GK (15Gy at the 50% isodose line) (Figure 1D). The patient continues to survive over two years after her first GK treatment. Her most recent MRI shows concern for radiation necrosis in the left middle frontal gyrus lesion with peritumoral edema around the left motor cortex (Figure 1E-1F). The patient has right upper extremity weakness (4/5 strength), and is on palliative systemic chemotherapy. The patient is on corticosteroids to manage the edema from radiation necrosis. There is no plan for any further treatments to the brain.

**Figure 2:**
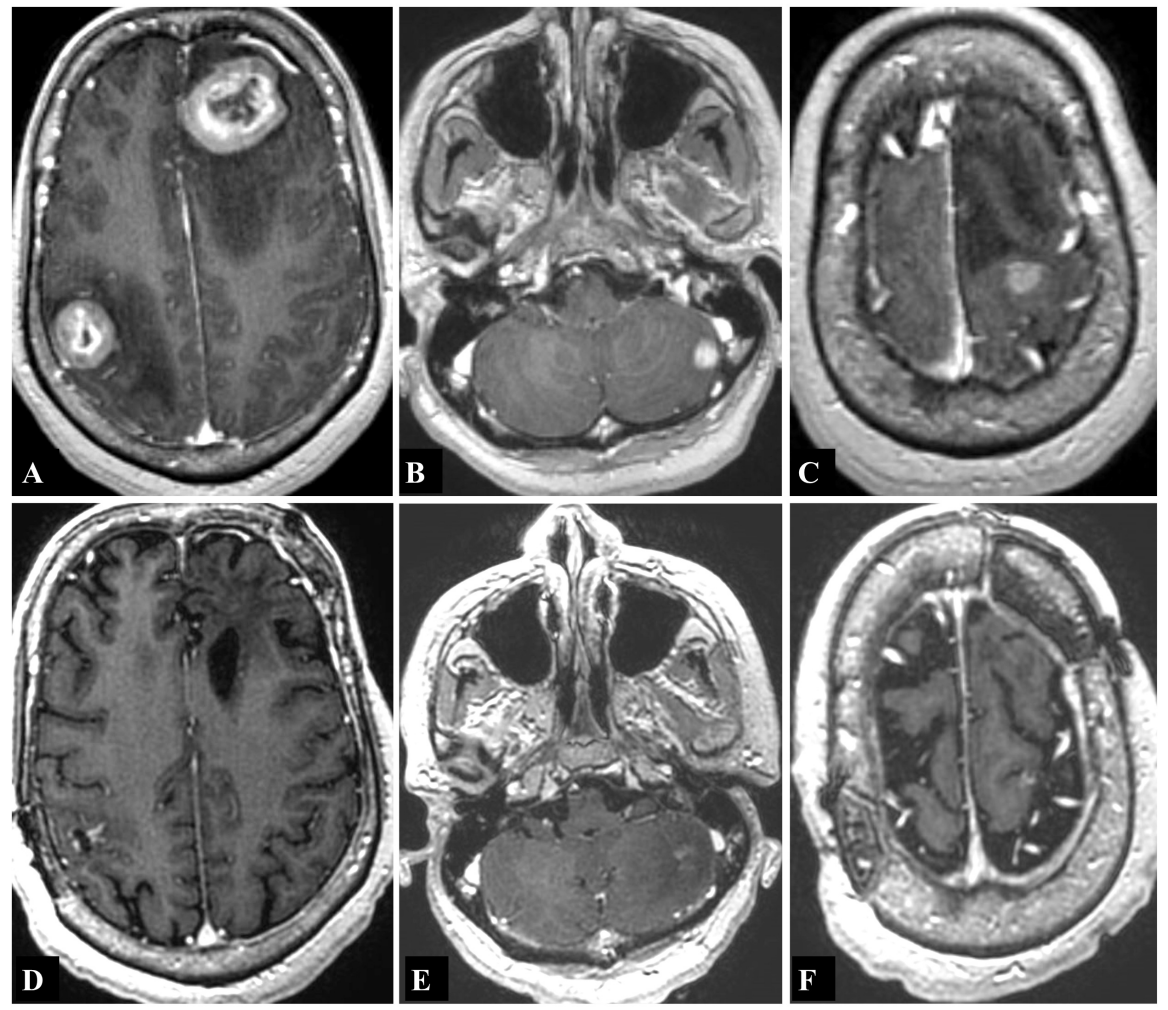
T1-weighted magnetic resonance imaging (MRI) with contrast showing contrast-enhancing lesions Lesions seen in the (A) right postcentral gyrus (2.35 cm in diameter) and left anterior superior frontal gyrus (3.5 cm), (B) left lateral cerebellar hemisphere (<1 cm), and (C) left precentral gyrus (<1 cm). (D,E,F) Most recent T1-weighted MRI with contrast obtained 32 months after first intracranial treatment demonstrates no new lesions and stable appearance of all treated lesions.

B. Case 2:

The patient is a 45-year-old female with a two-year history of breast cancer (infiltrating ductal carcinoma; estrogen and progesterone receptor positive). She presented with simple partial seizure of the left upper extremity followed by left hand weakness (3/5 strength). MRI of the brain with contrast demonstrated enhancing lesions in the right postcentral gyrus (2.35 cm diameter), with peritumoral edema extending to the central gyrus, and in the left anterior superior frontal gyrus (3.5 cm diameter) (Figure 2A). Lesions were also observed in the left lateral cerebellar hemisphere (less than 1 cm) and in the left precentral gyrus (less than 1 cm) (Figure 2B-2C). The patient underwent bilateral craniotomies under a single anesthesia setting for resection of the lesions in the right postcentral gyrus and left anterior superior frontal gyrus. The patient did not have any more seizures and she was full strength postoperatively. The postoperative MRI demonstrated complete resection of the tumor. On postoperative day 21, the patient underwent GK to both resection cavities (15Gy at the 50% isodose line to each lesion) to the left cerebellar lesion (24Gy at the 50% isodose line), and to the left precentral gyrus lesion (24Gy at the 50% isodose line). A follow-up MRI obtained 15 months after GK demonstrated excellent tumor control: no new lesions were identified, the surgically resected lesions were without recurrence, the left precentral gyrus lesion had completely resolved, and the left cerebellar lesion demonstrated signs of radiation necrosis. The patient has continued to survive four years after her diagnosis of breast cancer and 32 months after treatment of her brain tumors. She is currently on levetiracetam for seizure disorder and has complaints of headache managed by pain medications. Her most recent MRI shows no new brain lesions and a stable appearance of all treated lesions (Figure 2D).

**Figure 3:**
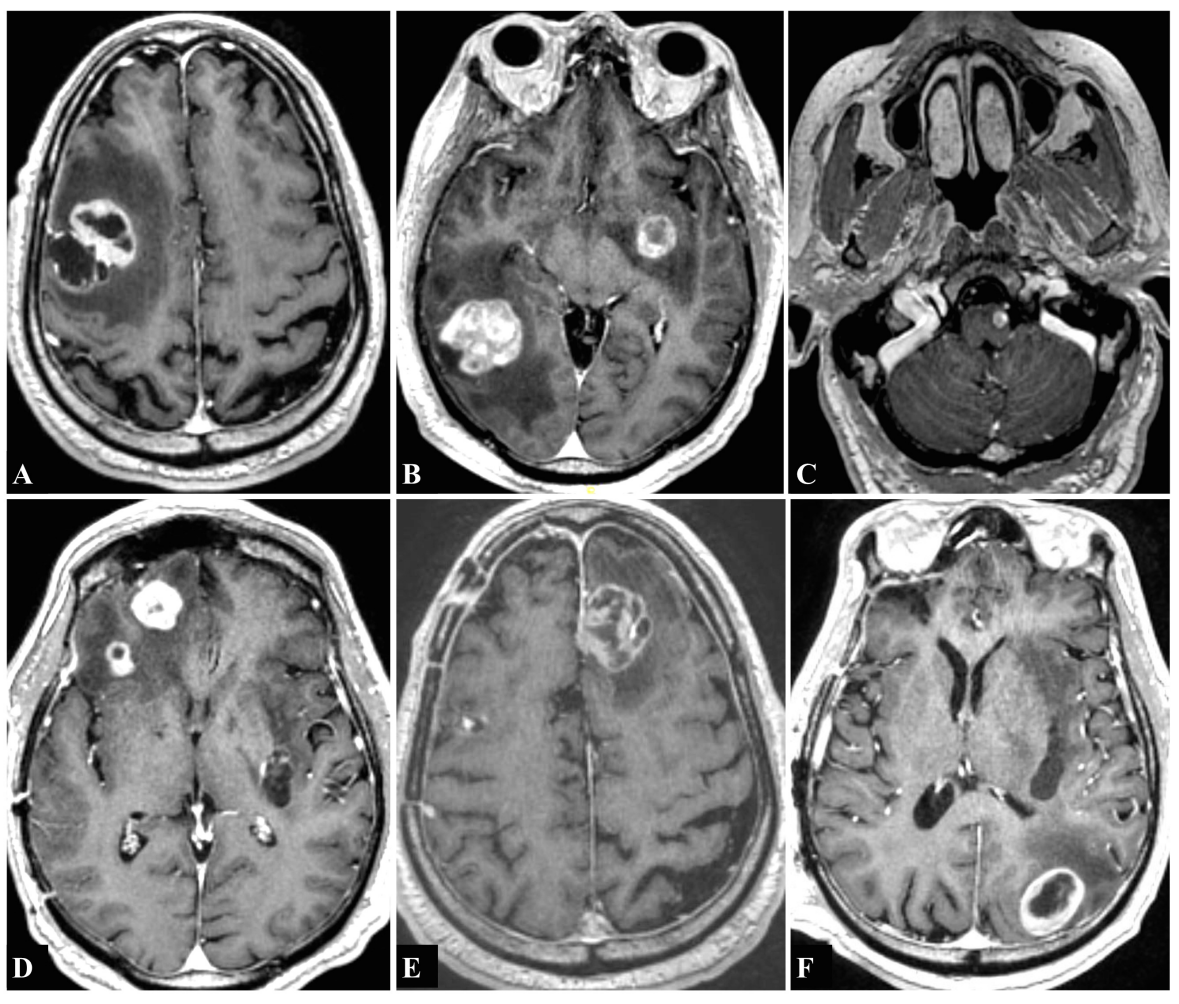
T1-weighted magnetic resonance imaging (MRI) with contrast showing contrast-enhancing lesions Lesions seen in the (A) right middle frontal gyrus (3.5 cm in diameter), (B) left basal ganglia (1.85 cm) and right posterior temporal lobe (4 cm), (C) left medulla (<1 cm), (D) right orbitofrontal cortex (2 cm and 1.25 cm), (E) left parasagittal frontal cortex (2.45 cm), and (F) left parietal cortex (2.4 cm).

C. Case 3:

The patient is a 60-year-old male with a recent diagnosis of renal cell carcinoma who presented to us with left upper extremity weakness and loss of dexterity. His strength exam was 4+/5 strength in his left biceps and triceps and 4/5 in his left hand. The MRI of the brain with contrast demonstrated a single lesion in the right middle frontal gyrus (3.5 cm in diameter) (Figure 3A). The patient underwent microsurgical resection of this right-sided lesion. Postoperative MRI showed complete resection of the lesion, and the patient’s strength improved to 4+/5. Three weeks later, the patient underwent GK to the postoperative resection cavity (16Gy at the 50% isodose line). Seven months later, a follow-up MRI showed complete resolution of the surgically resected right frontal lesion, but also showed new lesions in the left basal ganglia (1.85 cm in diameter) and in the right posterior temporal lobe (4 cm in diameter) (Figure 3B). The patient underwent microsurgical resection of the right temporal mass. The patient underwent GK to the right temporal postoperative cavity (15Gy at the 50% isodose line) and to the left basal ganglia lesion (24Gy at the 50% isodose line). Three months later, the patient had a follow-up MRI, which showed a new lesion in the left medulla, which was treated with GK (15Gy at the 50% isodose line) (Figure 3C). This MRI showed complete resolution of the surgically resected right temporal and right frontal lesions, and showed signs of radiation necrosis in the left basal ganglia lesion. A follow-up MRI four months later showed new lesions in the right orbitofrontal region (measuring 2 cm and 1.25 cm) and demonstrated recurrence of the previously resected right temporal lesion (Figure 3D). The patient underwent single craniotomy for the resection of both the new right frontal lesions, and three days later, the patient underwent another craniotomy for the resection of the right temporal recurrent lesion. Repeat GK of this recurrent temporal lesion was not performed. Three months later, the patient had a follow-up MRI, which showed a new left parasagittal frontal lesion (2.45 cm in diameter) (Figure 3E). The patient underwent microsurgical resection of this tumor, and three weeks later, the patient underwent GK (15Gy at the 50% isodose line) to the postoperative left parasagittal resection cavity. Four months later, the patient presented with seizures. MRI demonstrated a new left parietal lesion (2.4 cm) which was surgically resected (Figure 3F). Remarkably, this MRI showed excellent tumor control of all other surgically and GK-treated lesions. After undergoing six separate microsurgical resections and five separate sessions of GK SRS, the patient continues to survive over three years after his first microsurgical resection. He has not had any further microsurgical or GK treatments. His seizures are well controlled on levetiracetam.

## Discussion

The cases are organized in increasing order of complexity to demonstrate the impact of an aggressive neurosurgical and radiosurgical approach in achieving excellent patient outcomes. The evidence-based rationale in treating each patient is discussed as follows:

A. Multiple metastases, limited in number (1-4) and small (<3 cm): illustrative case 1

The first patient had two brain metastases smaller than 3 cm, one in each cerebral hemisphere, and each located in the region of the motor cortex. The patient received Gamma Knife (GK) alone for treatment of her metastases. Multiple studies have demonstrated favorable results in patients with 1-4 metastases treated with stereotactic radiosurgery (SRS) with or without whole brain radiation therapy (WBRT). In order to evaluate the utility of SRS and WBRT in patients with multiple brain metastases, Kondziolka, et al. randomized patients with 2-4 brain metastases to receive WBRT alone or WBRT + SRS [1]. The study was stopped early at 60% accrual because of superior results in the WBRT + SRS group. The median survival time (MST) was higher in the WBRT + SRS group (11 months vs 7.5 months), although the difference was not statistically significant (p=0.22). However, the rate of local failure, defined as tumor progression or increase in clinical symptoms, at one year among surviving patients was 100% in the WBRT group versus 8% in the WBRT + SRS group. This study showed that patients with multiple metastases treated with WBRT + SRS alone had a lower rate of local failure with a possible survival benefit.

Luther, et al. performed a retrospective study to define the role of SRS alone for brain metastasis in or around the motor strip [7]. 94 patients with 96 tumors (2 patients had bilateral lesions) with metastases > 1.5 cm in the region of the motor cortex were treated with GK SRS alone. Motor function improved in 30 cases (31%), remained stable in 48 (50%), and worsened in 18 (19%). The median tumor volume in patients who improved or remained stable was 5.3 cm3, and the median tumor volume in patients who worsened was 9.2 cm3. This study demonstrated that SRS alone for brain metastasis around the motor strip is safe as long as the tumor volume is around 5 cm3. For larger tumors, the authors recommended microsurgical resection followed by SRS to the postoperative cavity. The excellent outcome following management of the first patient’s lesions around the motor cortex validates the findings of the Luther, et al. study.

B. Multiple metastases, limited (1-4) and large (> 3 cm): illustrative case 2

This patient underwent surgical resection of two lesions. In a single setting, the patient received GK to both postoperative cavities and to two other lesions. Recent randomized control trials have demonstrated better tumor control in patients undergoing surgical resection of brain metastasis followed by SRS to the postoperative cavity. In a randomized control trial published in August 2017, Mahajan, et al. randomized patients who had a complete resection of one to three brain metastases (maximum diameter < 4 cm) to receive SRS to the resection cavity or observation [8]. At one year, freedom from local recurrence was 43% in the observation group and 72% in the SRS group (p=0.015). In another randomized control trial, also published in August 2017, Brown, et al. investigated the impact of SRS versus WBRT on survival and cognitive outcome in patients with resected brain metastasis [9]. Patients who underwent resection of single brain metastasis (maximum diameter < 5 cm) were randomized to receive postoperative SRS or WBRT. Although there was no difference in overall survival between the two groups, the rate of cognitive decline at six months was lower in patients who received SRS versus WBRT (52% vs. 85%, p<0.00031). The study concluded that SRS has a lower rate of cognitive decline with a more tolerable toxicity profile than WBRT in the postoperative setting. The two randomized control trials make a strong argument for adjuvant SRS to be the standard of care for patients with resected brain metastasis.

C. Multiple metastases, extensive (≥ 5), any size: illustrative case 3

The third patient in this series is the most complex. He received microsurgical resection followed by GK, which was repeated when new tumors were identified distant to the sites of initial metastasis.

Patients with extensive metastatic lesions are challenging to manage, and many physicians prefer to treat these patients with WBRT alone. However, studies have shown the benefit of SRS and surgery in treating patients with extensive metastases. In a prospective observational study, Yamamoto, et al. demonstrated that SRS alone as the initial treatment for patients with 5-10 brain metastases is non-inferior to that of patients with 2-4 metastases, with an MST of 10.8 months for both groups [6]. Patients included in the study had tumors <3 cm in largest diameter with a total cumulative tumor size <15 cc.

The role of surgical resection of multiple brain metastases is documented by retrospective studies. Bindal, et al. performed a retrospective review of 56 patients who underwent resection of multiple brain metastases [5]. The patients were divided into three groups: Group A (30 patients) had multiple metastases with one or more lesions left unresected, Group B (26 patients) had multiple metastases with resection of all lesions, and Group C (26 patients) had surgical resection of single metastasis. MST was six months for Group A, 14 months for Group B, and 14 months for Group C. There was a statistically significant difference in survival between Groups A and B, and Groups A and C, but not between Groups B and C. The recurrence rate was 31% in Group B and 35% in Group C. In Group B, a total of 44 craniotomies were performed to resect 55 lesions. The authors presented one of the first series demonstrating equivalent outcomes following surgical resection of single versus multiple metastases regardless of the number of craniotomies as long as all the lesions are resected completely.

Nevertheless, the problem of distant failure is a concern in patients with such overwhelming tumor burden, which can range from 22%-90% in the form of new metastasis [10]. Chang, et al. have noted that the probability of development of new metastasis is significantly higher in patients with more than 15 metastasis [10].

## Conclusions

Traditionally, physicians have hesitated to treat intracranial disease in patients with multiple brain metastases due to their perceived poor prognosis. Recent studies offer promising evidence that aggressive microsurgical and radiosurgical management may lead to excellent patient outcomes. The three cases discussed in this report are illustrative examples demonstrating excellent outcomes following treatment with evidence-based microsurgical and radiosurgical approaches.
